# Antithrombotic therapy after mitral valve repair: VKA or aspirin?

**DOI:** 10.1007/s11239-018-1724-0

**Published:** 2018-08-21

**Authors:** Sake J. van der Wall, Jules R. Olsthoorn, Samuel Heuts, Robert J. M. Klautz, Anton Tomsic, Evert K. Jansen, Alexander B. A. Vonk, Peyman Sardari Nia, Frederikus A. Klok, Menno V. Huisman

**Affiliations:** 1 Department of Thrombosis and Hemostasis, Leiden University Medical Centre, Albinusdreef 2, 2300 RC Leiden, The Netherlands; 2 Department of Cardio-Thoracic Surgery, Maastricht University Medical Centre, Maastricht, The Netherlands; 3 Department of Cardio-Thoracic Surgery, Leiden University Medical Centre, Leiden, The Netherlands; 4 Department of Cardio-Thoracic Surgery, VU University Medical Centre, Amsterdam, The Netherlands

**Keywords:** Mitral valve repair, Mitral valve annuloplasty, Antithrombotic therapy, Thromboembolism, Bleeding

## Abstract

The optimal antithrombotic therapy following mitral valve repair (MVr) is still a matter of debate. Therefore, we evaluated the rate of thromboembolic and bleeding complications of two antithrombotic prevention strategies: vitamin K antagonists (VKA) versus aspirin. Consecutive patients who underwent MVr between 2004 and 2016 at three Dutch hospitals were evaluated for thromboembolic and bleeding complications during three postoperative months. The primary endpoint was the combined incidence of thromboembolic and bleeding complications to determine the net clinical benefit of VKA strategy as compared with aspirin. Secondary objectives were to evaluate both thromboembolic and bleeding rates separately and to identify predictors for both complications. A total of 469 patients were analyzed, of whom 325 patients (69%) in the VKA group and 144 patients (31%) in the aspirin group. Three months postoperatively, the cumulative incidence of the combined end point of the study was 9.2% (95%CI 6.1–12) in the VKA group and 11% (95%CI 6.0–17) in the aspirin group [adjusted hazard ratio (HR) 1.6, 95%CI 0.83–3.1]. Moreover, no significant differences were observed in thromboembolic rates (adjusted HR 0.82, 95%CI 0.16–4.2) as well as in major bleeding rates (adjusted HR 1.89, 95%CI 0.90–3.9). VKA and aspirin therapy showed a similar event rate of 10% during 3 months after MVr in patients without prior history of AF. In both treatment groups thromboembolic event rate was low and major bleeding rates were comparable. Future prospective, randomized trials are warranted to corroborate our findings.

## Highlights

- The appropriate antithrombotic therapy following MVr is still a subject of controversy
- Consecutive patients who received either VKA or aspirin strategy were evaluated for thromboembolic and bleeding complications occurring within three months after MVr
- VKA and aspirin therapy showed a similar event rate of 10% during the first three months after MVr in patients without prior history of AF
- The choice of antithrombotic treatment should be individualized based on patient-specific considerations, such as risk factors for AF, compliance with treatment and frailty

## Introduction

Mitral valve repair (MVr) is recognized as the gold standard for degenerative mitral regurgitation. Compared to mitral valve replacement, repair results in improved survival, better preservation of postoperative left ventricular function and avoidance of the need for long-term anticoagulation treatment [1, 2]. The risk of thromboembolic events following MVr varies from 0.4 to 1.6% per year, and reaches 2.5% during the first postoperative month, even with routine anticoagulation therapy [3, 4]. However, the appropriate antithrombotic therapy following MVr is still a subject of controversy. Recommendations from international guidelines for the postoperative antithrombotic management have been controversial [5–7], and are based on observational studies without conclusive results, or are provided without references supporting the recommendation [4, 8–11].

Consequently, antithrombotic prophylaxis for the prevention of thrombotic events early after MVr varies widely among cardio-thoracic surgeons with a vitamin K antagonist (VKA) prescription varying from 46 to 64% in patients with sinus rhythm [12, 13]. The risk of thromboembolism secondary to a high incidence of new onset atrial fibrillation (AF) postoperatively and the thrombogenic tendency of the non-endothelialized repair components could motivate surgeons and cardiologists to prescribe VKA therapy for the first months after MVr [14]. However, evidence is limited and more accurate knowledge of the postoperative antithrombotic treatment is required. Based on recent literature and anecdotal reports, we hypothesized that VKA treatment is associated with an increased risk of major bleeding events and no reduction in thromboembolic events [18].

We set out to perform a retrospective observational study to evaluate the rate of thromboembolic and bleeding and complications of two antithrombotic prevention strategies—one with VKA and one with aspirin—occurring within the first three postoperative months.

## Materials and methods

### Study design and patients

This study was a retrospective observational multicentre cohort study of consecutive adult patients who underwent MVr, to evaluate thromboembolic and bleeding complications of two antithrombotic strategies, VKA and aspirin. Data were collected from the databases of the departments of cardiothoracic surgery of the Leiden University Medical Centre (LUMC), VU University medical centre (VUmc) and Maastricht University Medical Centre (MUMC). Patients who underwent a first MVr with or without concomitant tricuspid valve repair (TVr) between 2004 and 2016 in these three centres were eligible. The post-operative care of these patients often took place in one of 16 affiliated regional hospitals, in which all postoperative medical files were scrutinized for primary and secondary endpoints. Patients were excluded when they underwent other concomitant cardiac procedures than TVr, had previous cardiac surgery or were diagnosed with AF preoperatively. Other concomitant procedures were excluded because these lead to more heterogeneous patient groups. The institutional review board of the LUMC, VUmc and MUMC approved the study protocol and waived the need for informed consent due to the observational design.

### Procedures and treatment

MVr was performed at the department of cardiothoracic surgery at the LUMC, VUmc or MUMC and involved implantation of an annuloplasty ring (Edwards Physio I or II mitral ring, Carpentier-Edwards Classic Annuloplasty Ring or Duran AnCore Ring for MVr, and Edwards MC3 tricuspid ring or Carpentier-Edwards Classic Annuloplasty Ring in case of concomitant TVr, *Edwards Lifesiences*/*Medtronic, USA*), and various concomitant techniques (leaflet resections, artificial chorda tendinae implant, chordal transposition, or edge-to-edge technique).

Group A comprised patients from the LUMC and VUmc hospitals, in which therapeutic doses of low-molecular-weight heparin (LMWH) nadroparin were given on the first postoperative day at 7600 IU/day for patients < 50 kg, 11.400 IU/day for patients 50–70 kg, 15.200 IU/day for patients 70–100 kg and 19.000 IU/day for patients > 100 kg simultaneously with VKA. Treatment with nadroparin was continued until a VKA reached therapeutic levels, as shown by an international normalized ratio (INR) > 2.0 on two consecutive days. VKA therapy was maintained for 6–12 weeks postoperatively and then discontinued at the discretion of the referring cardiologist and occasionally switched to aspirin. The target INR during VKA treatment was 2.0–3.0.

Group B consisted of patients from the MUMC hospital, in which prophylactic doses of nadroparin were started on the first postoperative day at 3750 IU/day for patients < 80 kg, 5700 IU/day for patients 80–100 kg and 7600 IU/day for patients > 100 kg simultaneously with aspirin 80 mg once daily which was continued lifelong in patients with sinus rhythm. Nadroparin was stopped as soon as the patient was fully mobilized. In case of postoperative new onset AF that sustained for more than 24 h, nadroparin and VKA were started analogous to the antithrombotic strategy used in the LUMC and VUmc.

### Study endpoints

The primary endpoint of this study was the combined incidence of thromboembolic and major bleeding complications 3 months following MVr. This double endpoint was the basis for determining the net clinical benefit of VKA as compared with aspirin. Since we anticipated a high incidence of postoperative new onset AF, we also compared the primary endpoint in patients who did not develop AF during follow-up as well as in patients who received treatment according to the preferred strategy.

Secondary objectives were to evaluate the incidence rates of thromboembolic and major bleeding events separately and to identify predictors for bleeding and thrombotic complications. All thromboembolic and bleeding events were classified using the criteria for reporting mortality and morbidity after cardiac valve interventions respectively and those of the International Society on Thrombosis and Haemostasis respectively [15, 16]. Thromboembolic and bleeding complications occurring on the first postoperative day were not taken into consideration because both antithrombotic therapies were started this day. All suspected bleeding events were independently adjudicated by two expert physicians (F.K. and M.V.) who were blinded to treatment assignment. Disagreement was resolved by consensus.

Predefined candidate predictors for thromboembolic and bleeding events were defined according to the documentation provided by the treating physician, e.g. age, sex, prior arterial or venous thromboembolism, prior PCI, hypertension, history of smoking, preoperative use of anticoagulation therapy, left ventricular ejection fraction (LVEF), concomitant TVr, repeat thoracotomy and new onset AF. The cause of death was verified by reviewing the pathology report. In case autopsy had not been performed, the likely cause of death was verified with the treating physician. All patients were followed and censored at a maximum follow up period of 3 months, the date of last chart documentation, reoperation or outcome events, whichever came first.

### Statistical analyses

Means (standard deviation [SD]) and medians (interquartile range [IQR]) to present baseline continuous baseline variables were used. For categorical variables, frequencies and percentages were used. Pearson’s *χ*^2^ test was used to compare the distribution of categorical variables, whereas the independent *t*-tests were used for normally distributed continuous variables. For analysis of primary and secondary objectives, cumulative incidences of bleeding and thromboembolic events of both antithrombotic strategies were estimated according to the Kaplan–Meier methods and presented with two-sided 95% confidence intervals (CI). A Cox proportional hazard model was used to compare both strategies, adjusted for age, gender, and baseline differences.

Backward conditional multivariate Cox-regressions analysis was used to evaluate possible predictors for thrombotic and bleeding events, using variables of clinical importance (age and gender) or that were identified to be relevant predictors (P < 0.1) in univariate analysis. Data were analyzed using SPSS version 23 (SPSS, Chicago, IL, USA). A *P*-value below 0.05 was considered to be significant.

## Results

### Patients

In the three participating cardiothoracic surgical centers, 809 patients underwent a first isolated MVr between 2004 and 2016. Of these patients, 340 (42%) were excluded for the following reasons: 109 did not receive treatment in one of the affiliated regional hospitals postoperatively (4.9%), 224 had preoperative AF (10%) and seven patients were lost to follow up (0.32%). The remaining 469 (21%) patients were included; 325 patients (69%) in group A and 144 patients (31%) in group B. The baseline characteristics of both groups are shown in Table 1. Their mean age was 61 (SD 12) and 280 patients (60%) were men. Patients in group A underwent concomitant TVr more frequently (22% vs. 4.9%). In group B, a LVEF below 40% and preoperative aspirin use were more present (9% vs. 3.8% and 27% vs. 18% respectively). A total of 220 patients (47%) developed new onset AF after surgery and 35 patients (7.5%) required a repeat thoracotomy.

**Table 1:** Baseline characteristics of 469 patients who underwent MVr

Patient characteristics	Group A: VKA (n = 325)	Group B: aspirin (n = 144)
Age at operation, mean ± SD	60 ± 13	62 ± 11
Male, n (%)	195 (60)	85 (59)
Prior ischemic stroke, n (%)	7 (2.2)	8 (5.6)
Prior MI, n (%)	12 (3.7)	4 (2.8)
Prior PCI, n (%)	11 (3.4)	5 (3.5)
Prior VTE, n (%)	11 (3.5)	2 (2.6)
LV ejection fraction < 40%, n (%)	12 (3.8)	13 (9)*
Diabetes, n (%)	17 (5.4)	5 (3.5)
Hypertension, n (%)	149 (47)	74 (51)
COPD, n (%)	29 (8.9)	15 (10)
History of smoking, n (%)	99 (31)	27 (19)
Preoperative anticoagulation use, n (%)		
VKA	12 (3.7)	4 (2.8)
Aspirin	57 (18)	39 (27)*
Clopidogrel	3 (0.90)	2 (1.4)
Dual AP	1 (0.30)	2 (1.4)
Active endocarditis at the moment of surgery, n (%)	24 (7.4)	9 (6.3)
Concomitant TVr, n (%)	72 (22)	7 (4.9)*

*SD* standard deviation, *MI* myocardial infarction, *PCI* percutaneous coronary intervention, *VTE* venous thromboembolic event, *LV* left ventricular, *VKA* vitamin K antagonist, *AP* antiplatelet, *TVr* tricuspid valve repair

*P-value below 0.05

### Antithrombotic treatment

**Fig. 1:**
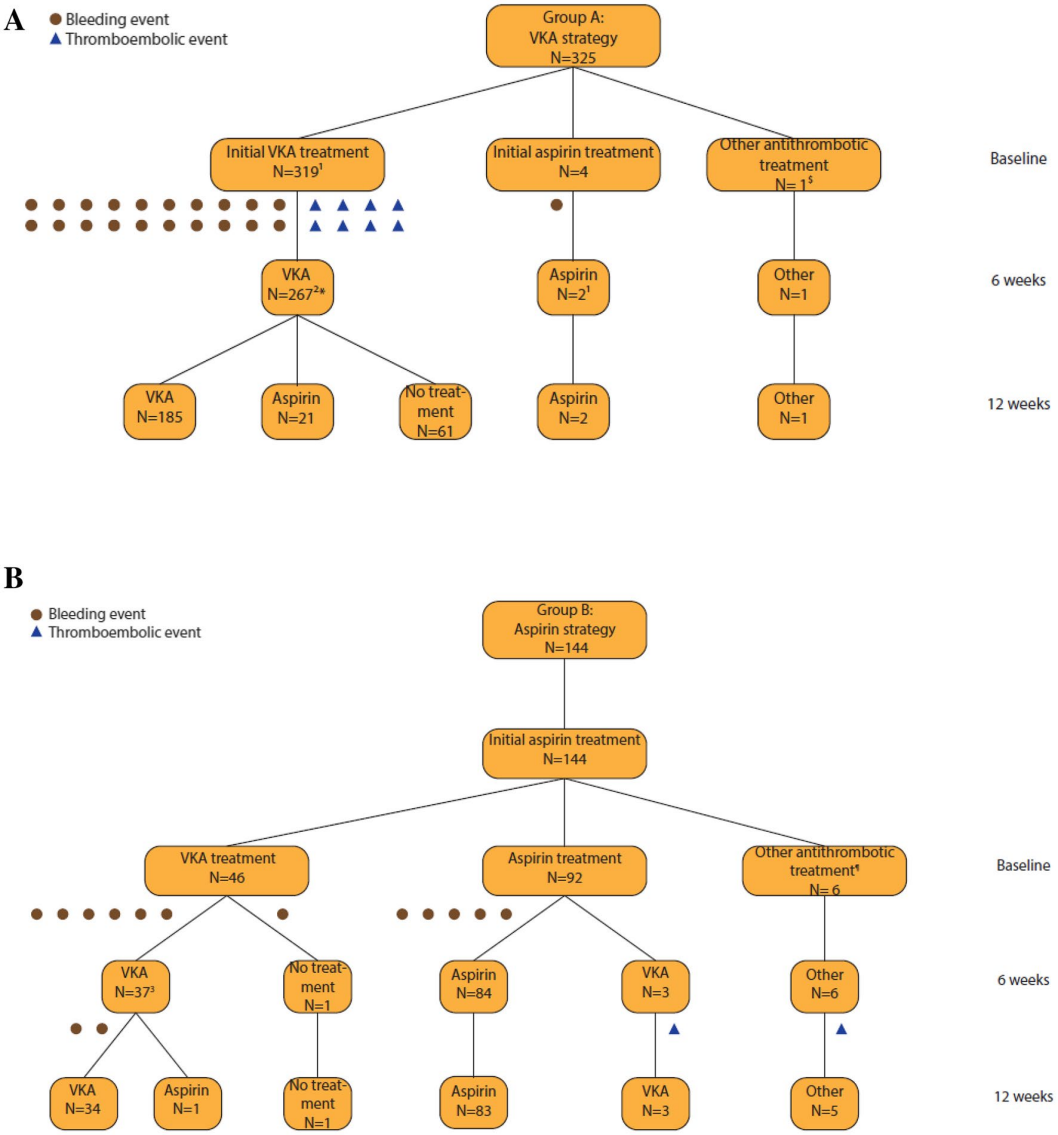
Flowchart of medication use and events of group A: VKA (**a**) group B: aspirin (**b**). ^1^1, ^2^8, ^3^1 patients censored for other reasons than study endpoints. *Data missing in 16 patients. ^$^2 patients treated with direct oral anticoagulant (DOAC), 4 patients with clopidogrel, ^¶^1 patient treated with low-molecular-weight heparin

Of the 325 patients in group A, 319 patients (98%) were treated with VKA therapy, four (1.2%) with aspirin therapy and one patient (0.31%) with LMWH (Fig. 1a). In group B, 92 of the 144 patients (64%) received aspirin, 46 patients (32%) VKA because of new onset AF and six patients (4.2%) received other antithrombotic therapy than VKA or aspirin (Fig. 1b). Twenty-three patients (25%) in group B, who received initial aspirin therapy, experienced a single episode of new onset AF.

### VKA versus ASA

Table 2 shows the incidence of thromboembolic and bleeding events in each study group. The primary end point of the study—the composite of thromboembolic and bleeding events—was reached in 29/325 patients in group A (cumulative incidence 9.2%, 95%CI 6.1–12) and in 16/144 patients in group B (cumulative incidence 11%, 95%CI 6.0–17; adjusted hazard ratio (HR): 1.6, 95%CI 0.83–3.1). The composite of thromboembolic and bleeding events in patients without new onset AF occurred in 14/177 patients (cumulative incidence 8.2%, 95%CI 4.1–12) in group A and 5/72 patients in group B (cumulative incidence 8.1%, 95%CI 2.0–14.2; adjusted HR 0.97, 95%CI 0.32–2.9). In patients who received initial treatment according to the preferable strategy, 28/319 patients experienced the primary endpoint in group A and 6/92 patients in group B during the first 3 months, for a cumulative incidence of 9.0% (95%CI 5.9–12) and 6.6% (95%CI 1.5–12) respectively (adjusted HR 0.90, 95% CI: 0.35–2.3).

**Table 2:** Clinical outcomes within 3 months after MVr

Bleeding events	Group A: VKA (N = 325)	Group B: aspirin (N = 144)
Major bleeding	21 (6.8)^a^	14 (9.1)
Site		
Chest	20	12
GI tract	0	1
Unknown	1	1
Fatal bleeding	1	1
Thromboembolic events	8 (2.6)	2 (1.6)
Type		
Ischemic stroke	4	1
TIA	4	0
Left atrial thrombus	0	1
Fatal ischemic stroke	0	1

*GI* gastrointestinal, *TIA* transient ischemic attack, *MI* myocardial infarction, *DVT* deep venous thrombosis

^a^Numbers in parenthesis are cumulative incidence

### Thromboembolism and bleeding

A total of 8/325 thromboembolic events occurred in group A after a median duration of 9 days (IQ 3.3–15) and 2/144 in group B after a median duration of 50 days (IQR 45–50), for a respective cumulative incidence of 2.6% (95%CI 0.84–4.4) and 1.6% (95%CI 0–3.8; adjusted HR 0.82, 95%CI 0.16–4.2). 21/325 patients experienced a major bleeding in group A after a median duration of 12 days (IQR 8–15) and 14/144 patients in group B after a median duration of 11 days (IQR 4.8–20), for cumulative incidences of 6.8% (95%CI 4.1–9.5) and 9.1% (95%CI 4.2–14) respectively (adjusted HR 1.89, 95%CI 0.90–3.9). A total of 89% of the major bleeding events were pericardial tamponades, of which two were fatal (one in each group).

### Other observations

During the study period, four patients died (cumulative incidence 0.9%, 95%CI 0–1.9), of whom two died in group A and two in group B. Causes of death were pericardial tamponades (two patients), ischemic stroke and cardiac arrest.

### Predictors for thromboembolism and major bleeding

Uni- and multivariate analysis of predictors for thromboembolic and major bleeiding events in patients who received initial treatment according to antithrombotic strategy are shown in Table 3. Multivariate analysis revealed that only concomitant TVr was independently associated with an increased risk of bleeding events (odds ratio (OR) 2.8, 95%CI 1.4–5.7) for both groups. For thromboembolic events, no independent predictors were found by multivariate analysis.

**Table 3:** Predictors for major bleeding and thromboembolic events in 469 patients who underwent MVr

Predictor	Major bleeding		TE	
	Univariate RR (95%CI)	Multivariate RR (95%CI)	Univariate RR (95%CI)	Multivariate RR (95%CI)
Age > 60	0.94 (0.48–1.8)		0.71 (0.21–2.4)	
Female	1.2 (0.64–2.4)		0.37 (0.78–1.7)	
Prior ischemic stroke	–		3.1 (0.39–25)	
Prior MI	0.84 (0.12–6.1)		–	
Prior PCI	0.80 (0.11–5.8)		–	
Prior VTE	1.1 (0.15–8.0)		–	
LV ejection fraction < 40%	2.2 (0.78–6.3)		–	
Diabetes	1.9 (0.59–6.3)		–	
Hypertension	1.2 (0.62–2.3)		2.3 (0.61–9.1)	
History of smoking	0.78 (0.36–1.7)		1.5 (0.46–5.3)	
New onset AF	1.7 (0.88–3.4)		1.1 (0.33–4.0)	
Concomitant TVr	2.8 (1.4–5.7)*	2.8 (1.4–5.7)*	2.3 (0.59–8.9)	
Active endocarditis	1.7 (0.6–4.8)		1.5 (0.19–11)	

*MI* myocardial infarction, *PCI* percutaneous coronary intervention, *VTE* venous thromboembolic event, *LV* left ventricular, *AF* atrial fibrillation, *TVr* tricuspid valve repair

*P-value below 0.05

## Discussion

VKA and aspirin therapy showed a similar event rate of 10% during the first 3 months after MVr in patients without prior history of AF. In both treatment groups thromboembolic event rate was low and major bleeding rates were comparable.

Nearly all bleedings occurred soon after surgery, particularly during the first 2 weeks after MVr. Interestingly, most of these were pericardial tamponades that required repeat thoracotomy. In contrast, the thromboembolic events occurred more dispersed throughout the first 3 months.

### VKA versus aspirin treatment

We chose a primary combined endpoint of thromboembolic and bleeding rates because both events would have a comparable prognostic effect as both represent an important cause of death and disability after heart valve surgery [17]. A comparison between VKA strategy (group A) and aspirin strategy (group B) revealed no difference in the combined outcome of thromboembolic and bleeding complications as well as for both outcomes separately occurring within 3 months after MVr. As expected, a third of the patients in group B could not follow the aspirin strategy because of new onset AF and received VKA treatment instead of aspirin therapy. Both of these group B treatment groups experienced major bleeding events to a similar extent. However, after exclusion of AF patients in the entire study population as well as analysing patients who received treatment according to the preferable strategy, again no difference in the combined endpoint was found, despite a group B population with solely aspirin use. Of note, three thromboembolic events in the VKA group occurred within the first 4 days during which VKA treatment still had not yet reached therapeutic levels. The observed 3-month cumulative incidence for thromboembolic events is in aligned with those reported by previous studies [4, 18]. The observed incidence of major bleeding events was slightly higher than described in previous reports, probably due to the adjudication process of postoperative pericardial tamponade [19, 20]. Pericardial effusion alongside signs of hemodynamic instability was adjudicated as a pericardial bleeding, whereas these events might not be considered as (major) bleedings in previous studies.

### Perspective of international guidelines

Recommendations from international guidelines are contradictory to our results, favouring either VKA or aspirin as postoperative thromboprophylaxis 3 months after MVr [5, 6, 21]. Three former retrospective studies have compared antiplatelet with anticoagulation therapy in patients after MVr [8, 19, 20, 22]. Two studies found no differences in stroke and bleeding rate of early VKA treatment compared with aspirin therapy, suggesting that VKA treatment might not be necessary [20, 22]. The largest study to date by Paparella et al. [19] found less bleeding and comparable arterial thromboembolic events in patients treated with aspirin 6 months following MVr. However, in contrast to our study, no data on AF were reported and assigned treatment was mainly chosen by the surgeons’ preference. A small study by Aramendi et al. [8]. found a beneficial effect of antiplatelet therapy in preventing thromboembolic events compared with VKA treatment with no increased risk of bleeding. Thus, these four studies suggest aspirin use after MVr. This contradicts the recommendation of VKA use over aspirin by the American College of Cardiology/American Heart Association (ACC/AHA) and European Society of Cardiology/European Association of Cardiothoracic Surgery (ESC/EACTS) guidelines [6, 21]. The ACC/AHA recommendations are based on one observational cohort study which found a high 30-day ischemic stroke incidence of 1.5%, despite VKA treatment [4, 21]. The ESC recommendation is provided without references, illustrating the paucity of information [6]. Since recommendations from guidelines are based on retrospective and underpowered studies, the optimal thromboprophylaxis after MVr remains controversial and a frequent matter of debate. However, based on the scarcity of data, our results might suggest a reassessment of the recommendations from international guidelines.

### Predictors

In our study, only concomitant TVr was found to be an independent predictor for major bleeding events. Concomitant TVr might have been a more difficult procedure with prolonged cardiopulmonary bypass duration, leading to dysfunction of platelets, which is associated with major cause of excessive bleeding in the early postoperative period [23, 24]. Other not predefined predictors, such as surgery duration, preoperative hematologic laboratory values and surgical techniques might also have contributed to the occurrence of early bleeding events. Consistently with earlier findings, no independent predictors were found for thromboembolic events [18].

### Clinical perspective

When considering the appropriate antithrombotic treatment after MVr, the thrombotic risk secondary to the endothelialization process and new onset AF could be a good rationale for physicians to prescribe VKA treatment. During the first three postoperative months, the exposure of circulating blood to non-endothelialized repair components can cause thrombus formation and even endocarditis, particularly due to a relatively slower blood flow in the left atrium compared to other parts of the heart. AF is a common postoperative cardiac arrhythmia after MVr occurring in approximately 24–35% of the patients, even after two postoperative weeks [14, 25]. In this study we found this incidence of new onset AF to be 47%. VKA treatment, however, has many disadvantages, including need for frequent laboratory monitoring, variability of dose response and drug and food interactions while in contrast aspirin does not require monitoring and dosage adjustments. Consequently, for practical reasons, aspirin might be preferable as antithrombotic treatment compared to VKA in patients with sinus rhythm. Therefore, the choice of antithrombotic treatment in patients without prior history of AF should be individualized based on patient-specific considerations, such as risk factors for AF, compliance with treatment and frailty. Despite the lack of prospective studies specifically evaluating treatment with direct oral anticoagulants (DOACs) in patients with mitral valve repair, subanalysis of DOAC AF trails have showed a similar overall efficacy and safety as compared with VKA in patients with valvular heart disease, including mitral valve repair [26]. However, international guidelines do not recommend the use of DOACs during the first three to six postoperative months in patients with AF [5–7].Future prospective randomized trials are warranted to provide conclusive results about DOAC treatment in the early postoperative phase after mitral valve repair in patients with and without AF.

### Strengths and limitations

The strength of this study is the large cohort of consecutive patients providing novel and clinically relevant data on the antithrombotic strategy after MVr. Moreover, the study population was rather homogeneous ,due to the exclusion of concomitant procedures that might lead to different patient groups (i.e. AF, other valve and coronary atherosclerotic surgery).

Our study had several limitations as well. First, a direct comparison between patients treated with VKA and aspirin would have been preferable but the high incidence of AF makes such a trial difficult to perform. A large number of patients would be required, in particular patients receiving aspirin. Second, antithrombotic treatment was not randomly allocated due to the retrospective study design. Third, no data was available on individual INR measurements and thus the time during which VKA treated patients were in therapeutic range is unclear. Fourth, we performed a multi-centre study with inherent perioperative variabilities. Ideally, future prospective, randomized clinical trials are warranted to provide evidence-based recommendations for the implementation of appropriate antithrombotic strategy after MVr.

VKA and aspirin therapy showed a similar event rate of 10% during 3 months after MVr in patients without prior history of AF. In both treatment groups thromboembolic event rate was low and major bleeding rates were comparable. Future prospective, randomized trials are warranted to corroborate our findings.
